# Ratiometric Fluorescent Biosensors for Glucose and Lactate Using an Oxygen-Sensing Membrane

**DOI:** 10.3390/bios11070208

**Published:** 2021-06-25

**Authors:** Hong Dinh Duong, Jong Il Rhee

**Affiliations:** School of Chemical Engineering, Chonnam National University, Yong-Bong Ro 77, Gwangju 61186, Korea; dinh70@jnu.ac.kr

**Keywords:** ratiometric fluorescent sensor, glucose sensor, lactate sensor, porphyrin dye, coumarin 6, glucose oxidase, lactate oxidase

## Abstract

In this study, ratiometric fluorescent glucose and lactate biosensors were developed using a ratiometric fluorescent oxygen-sensing membrane immobilized with glucose oxidase (GOD) or lactate oxidase (LOX). Herein, the ratiometric fluorescent oxygen-sensing membrane was fabricated with the ratio of two emission wavelengths of platinum meso-tetra (pentafluorophenyl) porphyrin (PtP) doped in polystyrene particles and coumarin 6 (C6) captured into silica particles. The operation mechanism of the sensing membranes was based on (i) the fluorescence quenching effect of the PtP dye by oxygen molecules, and (ii) the consumption of oxygen levels in the glucose or lactate oxidation reactions under the catalysis of GOD or LOX. The ratiometric fluorescent glucose-sensing membrane showed high sensitivity to glucose in the range of 0.1–2 mM, with a limit of detection (LOD) of 0.031 mM, whereas the ratiometric fluorescent lactate-sensing membrane showed the linear detection range of 0.1–0.8 mM, with an LOD of 0.06 mM. These sensing membranes also showed good selectivity, fast reversibility, and stability over long-term use. They were applied to detect glucose and lactate in artificial human serum, and they provided reliable measurement results.

## 1. Introduction

Glucose is an important compound involved in many metabolic pathways of living organisms. Therefore, many analytical methods and sensors for glucose detection have been developed and applied in various fields of life sciences [1,2,3,4,5]. At present, the most commercially successful application of biosensors has been in glucose biosensors for blood glucose analysis, i.e., in portable glucose meters used by diabetics [6]. However, glucose biosensors still face many obstacles to achieving clinically accurate glucose monitoring [7]. Among optical glucose sensors, fluorescent glucose sensors show advantages over conventional analytical techniques because fluorescence signals have clear recognition, high distinction, immunity to external disturbance, stability, and low noise [8]. In addition, the fluorescence probes of glucose sensors can detect glucose remotely by using an external light that can penetrate several centimeters into tissues as an excitation source [9,10]. Interactions between fluorescence probes conjugated to biomaterials through fluorescence resonance energy transfer (FRET) in the sensors can also produce selective measurements, reduce interference caused by light scattering in tissues, and correct photobleaching or fluorophore loss through diffusion or degradation [10,11]. Thus, even if a fluorescence-based glucose biosensor is suitable for noninvasive and in vivo detection, as well as continuous glucose monitoring, the development of appropriate fluorophores is more difficult than the development of a spectroscopic analysis method [12].

Lactate is also one of the key compounds in the metabolisms of living organisms; it is produced from anaerobic metabolisms of glucose in muscles. The concentration of lactate changes substantially depending on the condition of the human body (e.g., high intensity of exercise, pathology, etc.) [13]. Both high and low amounts of lactate can hinder physical activity. Therefore, in medical treatment with blood lactate, lactate measurement has long been used as an indicator of health and clinical status. Many lactate sensors, including electrochemical lactate sensors and optical lactate sensors, have been reviewed by L. Rassaei et al. [13] and K. Rathee et al. [14]. Several new concepts have recently been developed for the fabrication of lactate sensors for noninvasive measurements, such as wearable sensor systems to detect lactate in sweat [15], screen-printed lactate electrodes [16,17], self-powered lactate sensors [18], and test strip lactate sensors [19]. Similar to fluorescence glucose biosensors, fluorescent lactate biosensors are also of great interest because they show great benefits through the specific interaction between fluorescence dyes and substrates. However, finding stable and appropriate fluorescence materials for the fabrication of lactate biosensors under normal conditions has proven difficult and must be addressed in the near future.

In the context of the viewpoints mentioned above and based on the oxidation reactions of glucose and lactate in the catalysis of enzymes (Equation (1)), we developed ratiometric fluorescent glucose and lactate biosensors using a ratiometric fluorescent oxygen-sensing membrane and enzymes (glucose oxidase (GOD) and lactate oxidase (LOX)). The consumption rate of oxygen in Equation (1) corresponds to the concentration of glucose/lactate in the sample.

Glucose/Lactate + O_2_ − (GOD/LOX) → Oxidized products + H_2_O_2_ (1)

**Scheme 1:**
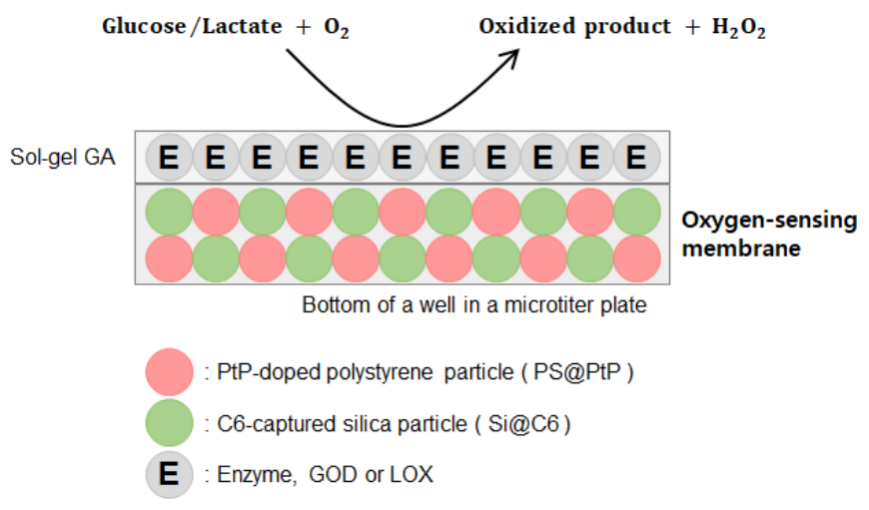
Layout of a ratiometric fluorescent biosensor.

To begin, the ratiometric fluorescent oxygen-sensing membrane was fabricated using platinum meso-tetra (pentafluorophenyl) porphyrin (PtP) doped in polystyrene particles (oxygen-sensing dye particles, PS@PtP) and coumarin 6 (C6) captured in silica particles (reference dye particles, Si@C6). The glucose-/lactate-sensing membrane consisted of both an oxygen-sensing membrane containing two dye particles as a transducer layer, and an enzyme (GOD or LOX) layer immobilized on the transducer layer using a functional sol-gel GA (mixture of 3-aminopropyltrimethoxysilane (APTMS) and 3-glycidoxypropyltrimethoxysilane (GPTMS)) as a supporting material (Scheme 1). The characteristics of the glucose- and lactate-sensing membranes were investigated using a ratiometric fluorescence calculation method, and biosensors were also used to detect the concentrations of glucose and lactate in human plasma.

## 2. Materials and Methods

### 2.1. Materials

Glucose, glucose oxidase (GOD, 26,820 unit(U)/g-solid, from *Aspergillus niger*), lactate oxidase (LOX, 41 units/mg-solid, from *Aerococcus viridans*), coumarin 6 (C6), tetraethyl orthosilicate (TEOS), 3-aminopropyltrimethoxysilane (APTMS), 3-glycidoxypropyltrimethoxysilane (GPTMS), bovine serum albumin (BSA), ethyl cellulose (EC), iron chloride, poly(vinyl pyrrolidone) (PVP, M.W. ≈ 55,000), styrene, 2,2′-azobisisobutyronitrile (AIBN), sodium dodecyl sulfate (SDS), L-ascorbic acid, dimethyl sulfoxide (DMSO), uric acid, urea, acetaminophen, and glycine were all purchased from Sigma-Aldrich Chemical Co. (Seoul, Korea). Pt(II) meso-tetra (pentafluophenyl) porphyrin (PtP) was obtained from Frontier Scientific Co. (Logan, UT, USA). Other chemicals, such as ammonium hydroxide (NH_4_OH), ethanol, tetrahydrofuran (THF), hydrochloric acid (HCl), sodium hydroxide, sodium phosphate, sodium chloride, and sodium bicarbonate were of analytical grade and used without further purification.

### 2.2. Preparation of PtP-Doped Polystyrene Particles (PS@PtP)

First, 25 mL of ethanol, 5 mL of deionized water, and 0.25 g of PVP were placed in a three-neck flask (100 mL) equipped with a condenser. The solution was then heated at 80 °C for 30 min, after which 2.5 mL of styrene and 100 µL of AIBN were sequentially added to the solution. Next, the polymerization was allowed to proceed for 6 h at 80 °C, and magnetic stirring was applied during the entire synthesis. Finally, the suspension of PS particles was cooled down to room temperature. The dispersed PS particles were then collected via centrifugation at 5000 rpm for 15 min, before being washed with ethanol three times and allowed to dry at room temperature.

A total of 200 mg of PS particles was added to 10 mL of 2% SDS solution and stirred for about 4 h to ensure a homogeneous dispersion of the particles. Next, 2.5 mL of 10 mM PtP in THF was added dropwise to the solution of PS particles under strong stirring for 12 h. The PS particles doped with PtP (PS@PtP) were collected via centrifugation at 5000 rpm for 15 min, then washed with distilled water three times and allowed to dry at room temperature.

### 2.3. Preparation of C6-Captured Silica Particles (Si@C6)

Coumarin-6-captured silica (Si) particles were prepared by a modified Stöber method [20]. First, C6 (3.5 mg) dissolved in 1 mL of DMSO was added into the solution of ethanol (10 mL) and distilled water (1 mL). Next, TEOS (4 mL) and NH_4_OH (2 mL) were added to this solution, after which it was stirred for 24 h at room temperature. The C6-captured silica particles (Si@C6) were collected by centrifugation at 12,000 rpm for 12 min. The Si@C6 were then further washed with distilled water and ethanol several times before being dried at room temperature.

### 2.4. Preparation of the PS@PtP*Si@C6 Membrane

A total of 50 mg of PS@PtP and 1.2 mg Si@C6 were mixed with 6 mL of 10 wt% EC in ethanol, and the mixture was stirred for 4 h before being coated on the bottom of a well in a 96-well microtiter plate (20 µL/well), then, finally, dried at 60 °C for 12 h.

### 2.5. Immobilization of Enzymes (GOD or LOX) onto the PS@PtP*Si@C6 Membrane

To begin, a layer of a given amount of enzyme (GOD or LOX) encapsulated in 10 µL sol-gel GA was coated onto the surface of the PS@PtP*Si@C6 membrane of each well in a 96-well microtiter plate. Sol-gel GA was formed through the hydrolyzation and polymerization of a mixture of APTMS and GPTMS in ethanol at a volumetric ratio of 6.5%:25%:68.5%, respectively, and the volume of HCl (37%) was 4% (*v*/*v*) of the sol-gel GA volume. After adding HCl, the sol-gel GA was kept at room temperature for 4 h before being used in the subsequent steps.

The optimal amount of GOD for the immobilization was tested with 10 U, 20 U, 50 U, 100 U, 150 U, and 200 U. Meanwhile, the optimal amount of LOX for the immobilization was tested with 0.2 U, 0.5 U, 1 U, 1.5 U, and 2 U. The immobilization efficiency of the enzymes onto the PS@PtP*Si@C6 membrane was calculated based on the Bradford assay. In addition, the PS@PtP*Si@C6 membranes immobilized with various amounts of GOD or LOX were measured with different concentrations of glucose or lactate. The sensitivity of the sensing membranes was evaluated based on the slope value (SI), i.e., the ratio of the fluorescence intensities at two emission wavelengths (*λ_em_* = 475 nm and 635 nm), with respect to the glucose or lactate concentration, to determine the optimal amount of GOD or LOX for immobilization. The kinetic parameters, i.e., maximal reaction rate (V_max_) and Michaelis-Menten constant (K_m_) of the immobilized enzymes, were determined from the Lineweaver–Burk plot based on the ratio of the fluorescence intensities at *λ_em_* = 475 nm and 635 nm.

### 2.6. Measurements of Glucose and Lactate

As shown in Scheme 1, the sensing membrane included a fluorescent oxygen-sensing layer (as a transducer) and an enzyme-immobilized layer, which was deposited on the bottom of one well in a 96-well microtiter plate. Afterwards, the sensing membrane was exposed to glucose or lactate solutions with different concentrations.

The responses of the sensing membranes to different glucose or lactate concentrations ranging from 0.1 mM to 10 mM were determined by a multifunctional fluorescence microtiter plate reader (Safire^2^, Tecan Austria GmbH, Austria). Data were collected from the fluorescence intensities of the sensing membrane at two emission wavelengths (*λ_em_* = 475 nm and 635 nm), with an excitation wavelength of 400 nm (*λ_em_* = 400 nm).

The reversibility of the sensing membranes was conducted with 2 mM glucose and distilled water for the glucose-sensing membrane, as well as with 1 mM lactate and distilled water for the lactate-sensing membrane. The microplate reader was set up for fluorescence measurements against time with an interval of 30 sec during measurements.

The effects of pH and temperature on the measurements of glucose or lactate were investigated at different temperatures (25, 27, 30, 33, and 35 °C) or in the range of pH 5.0–pH 9.0 with glucose or lactate concentrations varying from 0.1 to 10 mM. The long-term stability of the sensing membranes was evaluated with various glucose or lactate concentrations by measuring the fluorescence intensity obtained, both initially and after a certain time of use.

The interfering effects of some components contained in human serum, such as ions (Na^+^, Cl^−^, HCO_3_^−^, Fe^3+^) and albumin (BSA), on the glucose- and lactate-sensing membranes were investigated. The sensing membranes were measured with 145 mM Na^+^, 106 mM Cl^−^, 30 mM HCO_3_^−^, 1.625 mg/L Fe^3+^, and 5 g/dL BSA, with 1 mM glucose concentration or 1 mM lactate concentration.

### 2.7. Ratiometric Fluorescence Method and Data Analysis

For the sensing membranes, a ratiometric fluorescence method was used that was based on the ratio of the fluorescence intensities at two emission wavelengths (*λ_em_* = 475 nm (FI_475_) and 635 nm (FI_635_)) as follows:

R = FI_635_/FI_475_

The differences in the fluorescence intensities and slopes of the linear ranges of the sensing membranes at different interferences were assessed by one-way analysis of variance (ANOVA). Significant differences between samples were accepted with a *p*-value < 0.05. Statistical tests were performed using InStat software (vers. 3.01, Graph Pad Software Inc, San Diego, CA, USA).

## 3. Results and Discussion

### 3.1. The Operation Mechanism of the Sensing Membrane

The operation mechanisms of biosensors for the detection of glucose/lactate are usually based on the oxygen consumption or hydrogen peroxide production from the oxidation of glucose/lactate, as shown in Equation (1). In this work, the change in the concentration of oxygen is used to determine the concentration of glucose/lactate solutions. Herein, the oxidation of glucose or lactate by molecular oxygen will occur when the sensing membranes are exposed to glucose or lactate solutions under the catalysis of GOD or LOX to produce the oxidized products and hydrogen peroxide (H_2_O_2_). The consumption of oxygen in this oxidation reaction decreases the oxygen concentration in aqueous solutions and increases the fluorescence emission of the fluorescent oxygen-sensing layer in the sensing membrane. The fluorescent oxygen-sensing layer can measure oxygen concentrations through an increase or decrease in the fluorescence intensity in the absence or presence of oxygen, respectively. The change in the fluorescence intensity of the sensing membrane is proportional to the concentration of glucose or lactate solutions.

### 3.2. Properties of the Oxygen-Sensing Membrane (i.e., the PS@PtP*Si@C6 Membrane)

**Figure 1:**
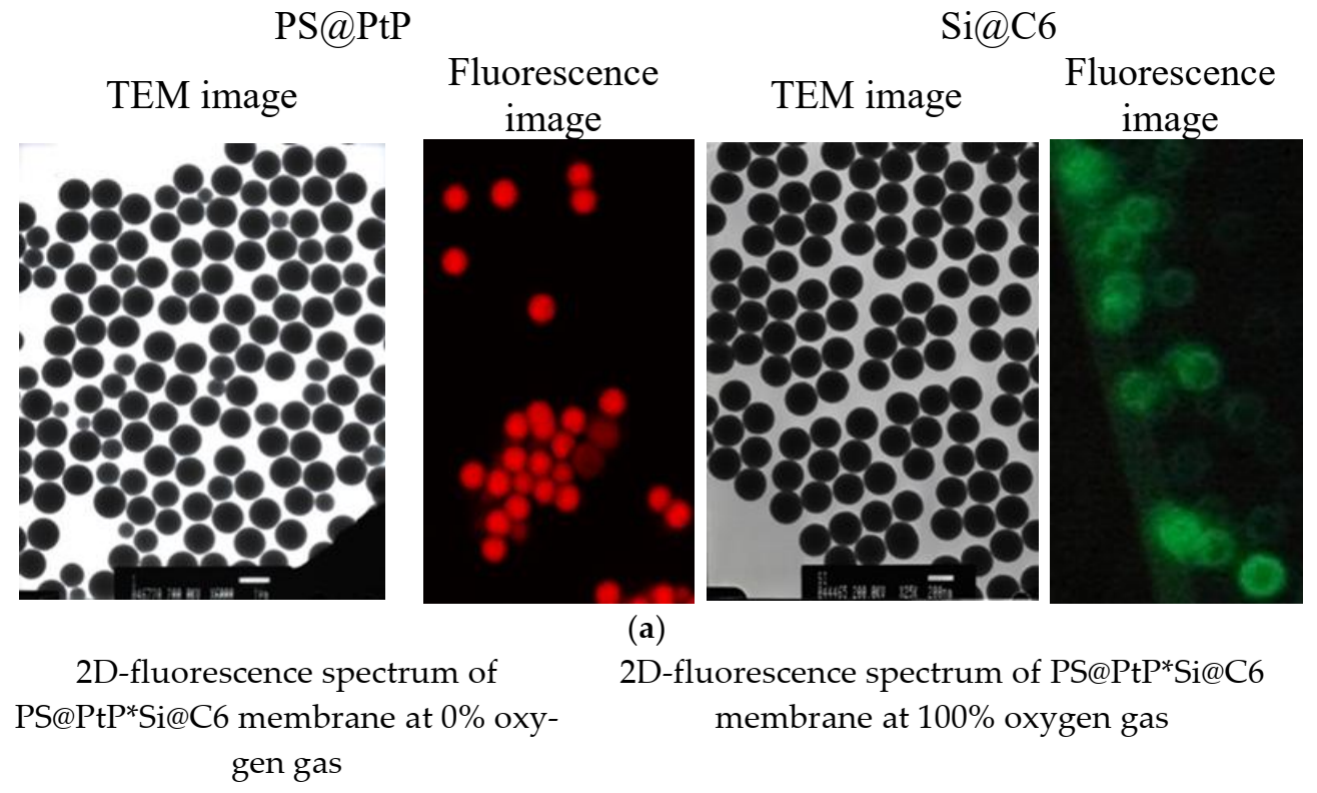

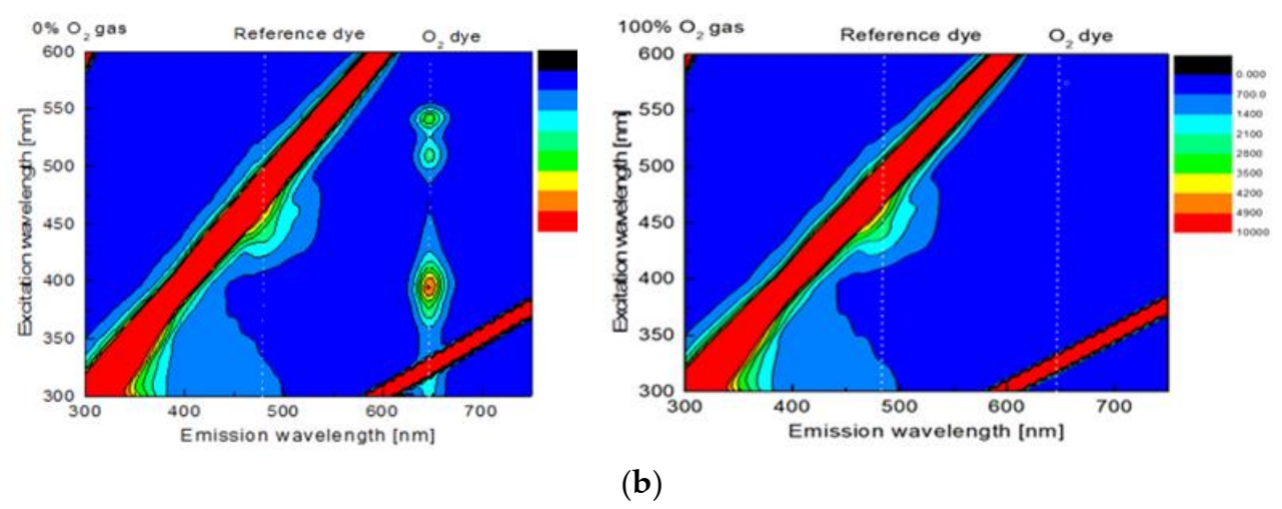
(**a**) TEM and fluorescence images of PS@PtP with a scale bar of 1 µm and Si@C6 with a scale bar of 200 nm; (**b**) 2D-fluorescence spectra of the PS@PtP*Si@C6 membrane to 0% and 100% oxygen gas.

As shown in Figure 1a, the synthesized PS particles and Si particles are spherical particles with respective diameters of approximately 1 µm and 0.2 µm. The particles containing the fluorescent dye, PtP or C6, also show red and green colors for PS@PtP and Si@C6, respectively. The PS@PtP*Si@C6 membrane shows emission band edges at λ_em_ = 635 nm for PtP (oxygen-sensing dye) and λ_em_ = 475 nm for C6 (reference dye) (Figure 1b). This membrane is highly sensitive to different oxygen concentrations. The fluorescence intensity of the PS@PtP in the membrane increased at 0% oxygen concentration (Figure 1b, left), and it was quenched when the oxygen concentration was increased up to 100% (Figure 1b, right). Meanwhile, the fluorescence intensity of the Si@C6 was stable at both low and high oxygen concentrations.

**Figure 2:**
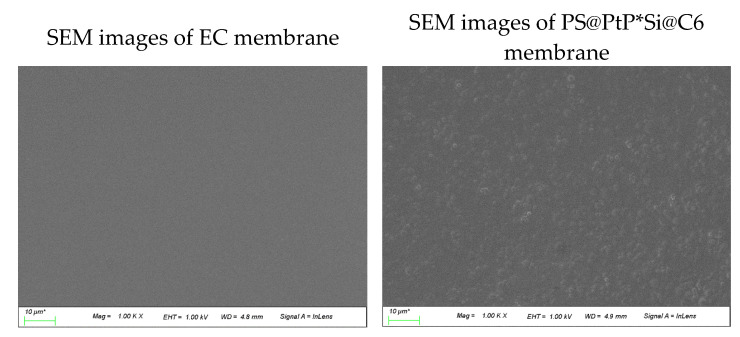

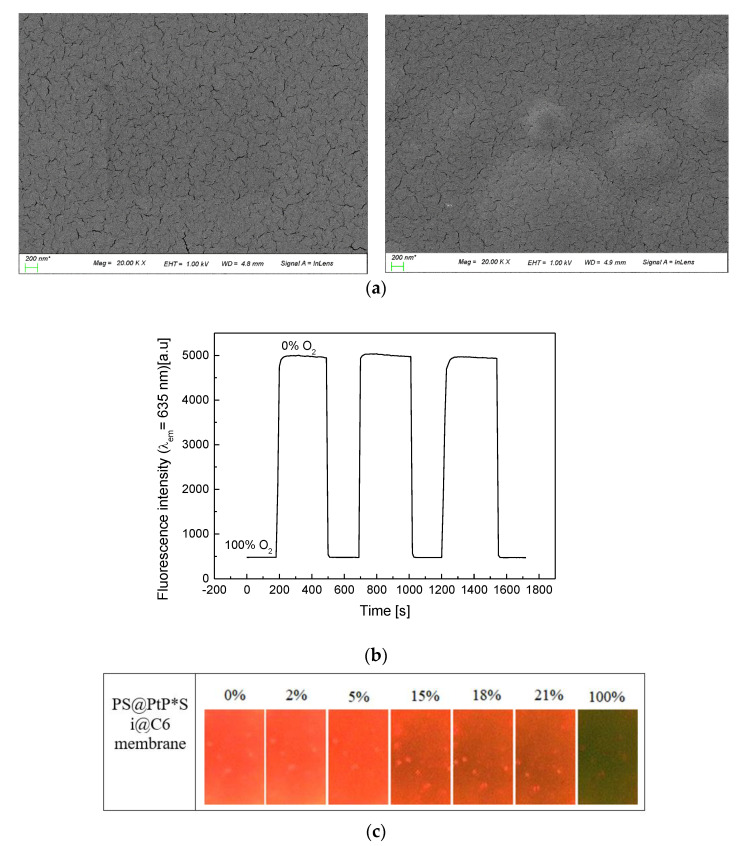
(**a**) SEM images of the EC membrane (left photos) and the PS@PtP*Si@C6 membranes (right photos) at different magnifications; (**b**) repeatability of the PS@PtP*Si@C6 membrane when exposed to concentrations of 100% and 0% oxygen gas; (**c**) photo images of the PS@PtP*Si@C6 membrane when exposed to different concentrations of oxygen gas.

According to the SEM images in Figure 2a (left), the surface of the EC membrane is smooth under low magnification (×1000) but shows many slits under high magnification (×20,000). This could be a main reason why the EC membrane is a good membrane for the penetration and convection of oxygen, and why it is widely used as a matrix for hosting many oxygen-sensing probes [21,22]. The photos in Figure 2a (right) also show the EC membrane containing both Si@C6 and PS@PtP under low and high magnifications. The PS@PtP and Si@C6 were completely encapsulated into the EC supporting membrane, but they were still in contact with oxygen through slits on/inside the EC membrane. Figure 2b shows that the PS@PtP*Si@C6 membrane has high sensitivity to different oxygen concentrations and good reversibility in the presence and the absence of oxygen, since RSD are 1.1 and 0.41% at 0 and 100% oxygen, respectively. These results confirm the high penetration and convection of oxygen inside the EC supporting membrane, as well as the high sensitivity of the PS@PtP at different oxygen concentrations.

Fluorescence quenching of the PtP dye occurs in the presence of 100% oxygen concentration, resulting in the clear emergence of the fluorescence of the reference dye (C6), as shown in Figure 2c. The red color of the PtP dye in the PS@PtP*Si@C6 membrane decreased with increasing oxygen concentration, and it showed the green color of the C6 dye at 100% oxygen concentration. This color change of the PS@PtP*Si@C6 membrane can be seen within seconds when exposed to different oxygen concentrations, and this is consistent with the data illustrated in Figure 2b.

### 3.3. Efficiency of Enzyme Immobilization on the PS@PtP*Si@C6 Membrane

**Scheme 2:**
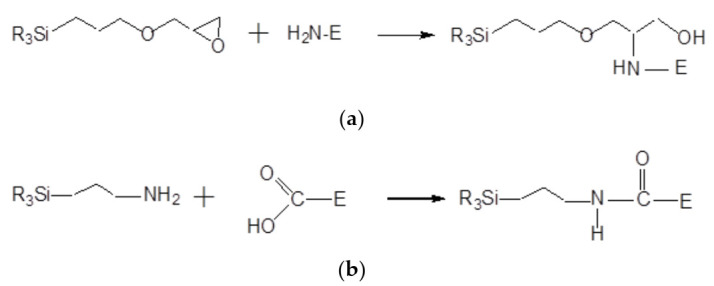
(**a**) Reaction of epoxy group of GPTMS and amine group of enzyme (E); (**b**) reaction of amine group of APTMS with carboxyl group of enzyme (E).

Enzymes can be immobilized onto the PS@PtP*Si@C6 membrane via sol-gel GA through two methods, as shown in reaction Scheme 2a,b [23,24]. That is, the epoxy group of GPTMS and the amine group of APTMS can covalently bind to the amine group and the carboxyl group of the enzymes, respectively. Therefore, large amounts of enzymes can be immobilized on/in sol-gel GA and the sensing membrane can remain stable for a long time of use.

**Figure 3:**
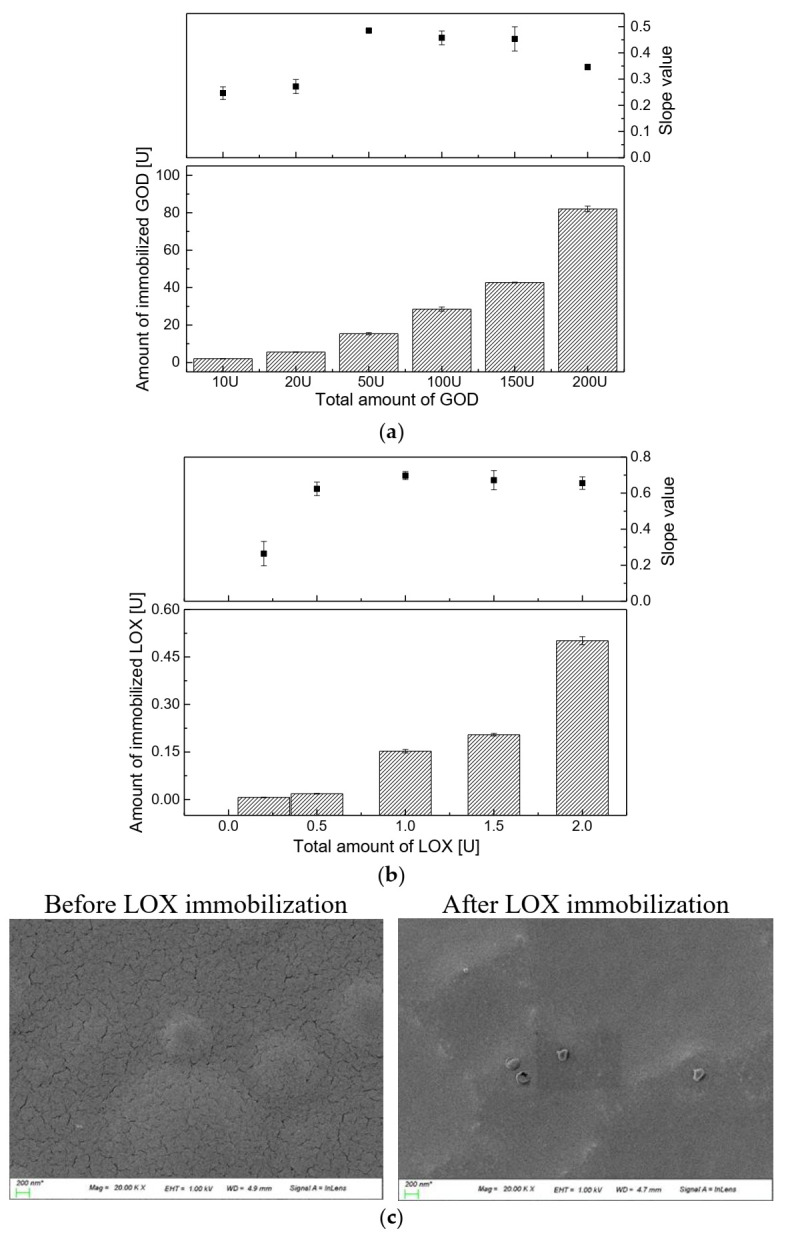
(**a**) Immobilization efficiency of different amounts of GOD, and slope values of the calibration curves of the glucose-sensing membranes in the glucose concentration range from 0 to 2 mM; (**b**) immobilization efficiency of different amounts of LOX, and slope values of the calibration curves of the lactate-sensing membranes in the lactate concentration range from 0 to 0.8 mM; (**c**) SEM images of the oxygen-sensing membrane before and after LOX immobilization.

According to the data collected from the Bradford protein assay, which is shown in Figure 3a (lower graph), the immobilization efficiency of GOD on the PS@PtP*Si@C6 membrane was increased with an increasing amount of GOD used. However, the slope value (SI) of the calibration curve of the glucose-sensing membranes immobilized with the initial GOD amount of 50 U (SI = 0.4849) was the highest in the concentration range of 0–2 mM glucose, while the SIs with 100 U GOD and 150 U were 0.4575 and 0.4533, respectively. Using lower amounts of GOD than 50 U could also lead to decreased SIs (Figure 3a, upper graph). According to the theory, too much immobilized enzyme may lead to a narrow detection range, or cause an obstruction of the transport of the analyte to contact with the transducer membrane. However, too little immobilized enzyme results in a slow reaction and response, as well as low sensitivity of the sensing membrane. Thus, for further measurements, it was confirmed that the initial GOD amount of 50 U was the most suitable amount for the immobilization of GOD onto the PS@PtP*Si@C6 membrane.

Regarding the immobilization of LOX (Figure 3b), it showed the same trend as the GOD immobilized onto the PS@PtP*Si@C6 membrane (Figure 3b, lower graph). The more LOX used, the more LOX that was immobilized. However, the highest SI of the calibration curve of the lactate-sensing membrane was obtained at an initial LOX amount of 1 U (SI = 0.6974). The initial LOX amounts of 0.5 U, 1.5 U, and 2 U could also be used for immobilization, but the SI of the calibration curve was slightly smaller. For the stability and economic efficiency of the lactate-sensing membrane, 1 U of LOX was chosen and used in this work. As shown in the SEM images of the PS@PtP*Si@C6 membrane after enzyme (LOX) immobilization (Figure 3c), the slits of the PS@PtP*Si@C6 membrane were filled through the incorporation of tightly covalent bonds between sol-gel GA and the enzymes (LOX). The sensing membranes containing sol-gel GA and the enzyme could somewhat reduce the connection between the PS@PtP*Si@C6 membrane and the oxygen in the surrounding environment. However, sol-gel GA is considered to be the best choice because its low viscosity produced a very thin layer.

### 3.4. Characterization of the Glucose-Sensing Membrane

**Figure 4:**
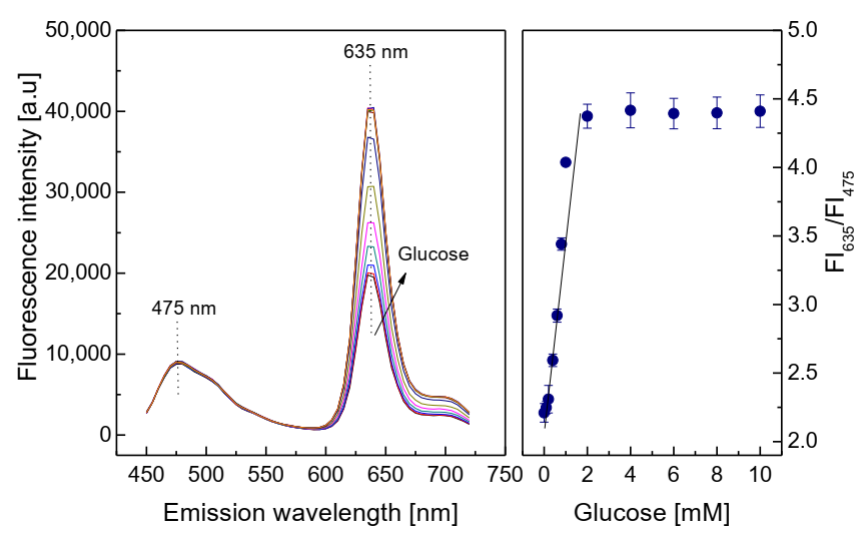
Response of the glucose-sensing membrane at different glucose concentrations, and calibration curve for glucose as determined by the ratio of FI_635_/FI_475_.

The consumption of oxygen in the oxidation reaction of glucose led to an increase in the fluorescence intensity of the PS@PtP in the glucose-sensing membrane, as shown in Figure 4. The fluorescence intensity of the glucose-sensing membrane at λ*_em_* = 635 nm increased with increasing glucose concentrations in the range of 0.1–10 mM; here, the linear detection range was chosen as 0.1–2 mM, with a limit of detection (LOD) of 0.031 mM. The activity of GOD immobilized on the sensing membrane was evaluated via Michaelis–Menten kinetics. The kinetic parameters were calculated from the ratio of the emission fluorescence intensities at λ*_em_* = 475 nm and 635 nm. The maximal reaction rate (V_max_) of 2500 mM/min and the Michaelis–Menten constant (K_m_) of 0.25 mM were obtained from the Lineweaver–Burk plot. Comparing the photo images of the response of the PS@PtP*Si@C6 membrane in Figure 2c with the graphs in the left of Figure 4 yields that the oxygen concentration in the detection of glucose could be varied in the range of 0–21%.

**Figure 5:**
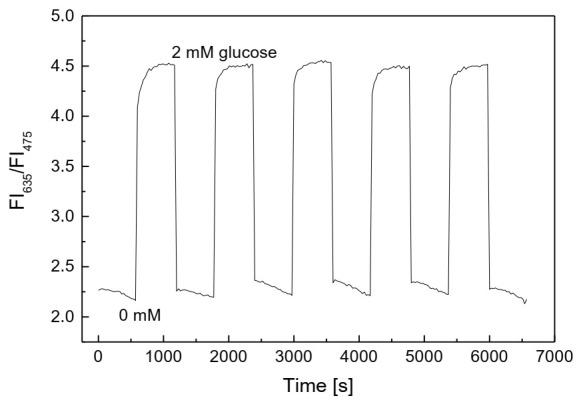
Reversibility of the glucose-sensing membrane repeatedly exposed at 0 and 2 mM glucose.

The PS@PtP*Si@C6 membrane showed very high sensitivity and repeatability when exposed to many repeated cycles of low and high concentrations of oxygen (Figure 2b). When GOD was immobilized onto the oxygen-sensing membrane, the reversibility of the membrane remained good when exposed to repeated cycles of glucose concentrations of 0 and 2 mM (Figure 5). The glucose-sensing membrane showed fast recovery between 0 and 2 mM glucose, with small values of RSD: 1.17% at 0 mM and 1.35% at 2 mM glucose. These results also indicated that the thickness of the second layer containing GOD did not significantly affect the contact between the PS@PtP*Si@C6 membrane and the oxygen in the glucose solution, since the saturation point was reached in the shortest time.

For the biosensors using enzyme, pH and temperature are the important parameters affecting the measurements. The solution pH can increase or decrease the activity of the enzyme in the sensor and, consequently, increase or decrease the efficiency of the catalytic reaction.

**Figure 6:**
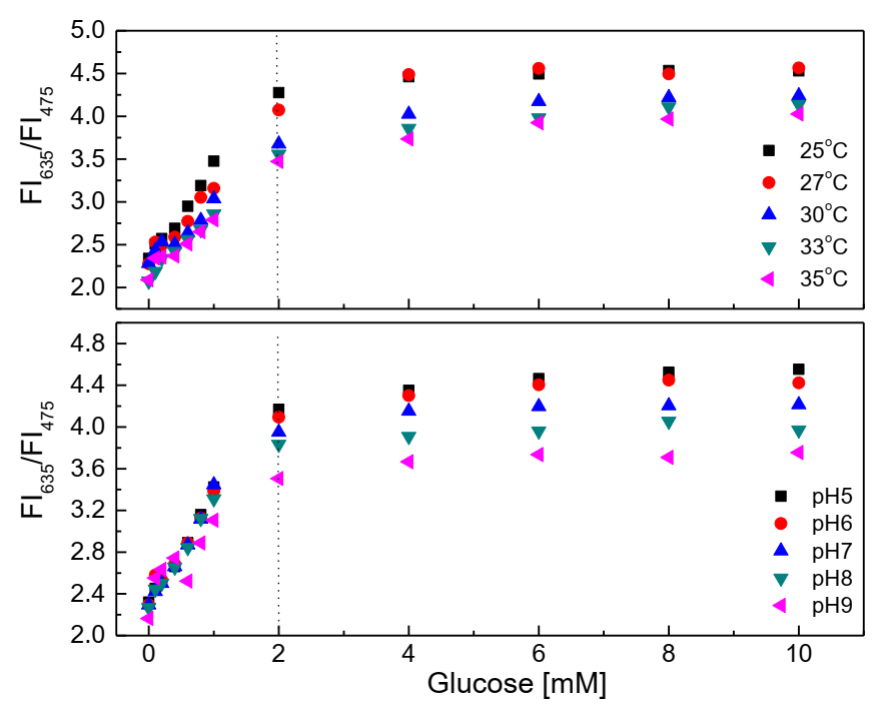
Response of the glucose-sensing membrane at different glucose concentrations in the temperature range of 25–35 °C and the pH range of 5–9.

Figure 6 (upper graph) shows the response of the glucose-sensing membrane with various glucose concentrations at different temperatures. The glucose-sensing membrane shows lower sensitivity at 35 °C than it does at other temperatures in the range of 25–33 °C. Decreasing the temperatures toward 25 °C could increase the sensitivity of the glucose-sensing membrane at all glucose concentrations. Our previous study found sol-gel GA to be a good material for heat transfer [25]. Moreover, it is easy to use a thin layer of the sol-gel GA to make the glucose-sensing membrane influenced by temperature; in fact, the higher the temperature, the higher the activity of the enzyme. However, in the present case, the sensitivity of the glucose-sensing membrane decreased at a high temperature. This may be due to the temperature dependence of the amount of oxygen in the glucose solution and the overlapping of fluorescence emission of the PtP dye when being excited at a high temperature. As shown in Figure 6 (lower graph), the glucose-sensing membrane preferred acidic medium (in the range from pH 5 to pH 6) to alkaline medium (pH 8 to pH 9).

The ratio of the fluorescence intensities at *λ_em_* = 475 nm and 635 nm of the glucose-sensing membrane did not change significantly at any glucose concentrations in the range of 0.1–10 mM in the pH range of 5–6. However, the sensitivity of the glucose-sensing membrane decreased with increasing pH from 7 to 8 and 9. Sol-gel GA has amine functional groups which prefer alkaline medium, but in this case, to obtain the transparent membranes, hydrochloric acid (HCl) was used to catalyze the polymerization of sol-gel GA, and the pH of sol-gel GA consequently became approximately pH 5. In addition, 10 w% ethyl cellulose solution also has a pH of approximately 5–6. These conditions may possibly match with the operation condition of GOD, thereby explaining the preferred operation of the glucose-sensing membrane in the pH range from 5 to 7.

**Figure 7:**
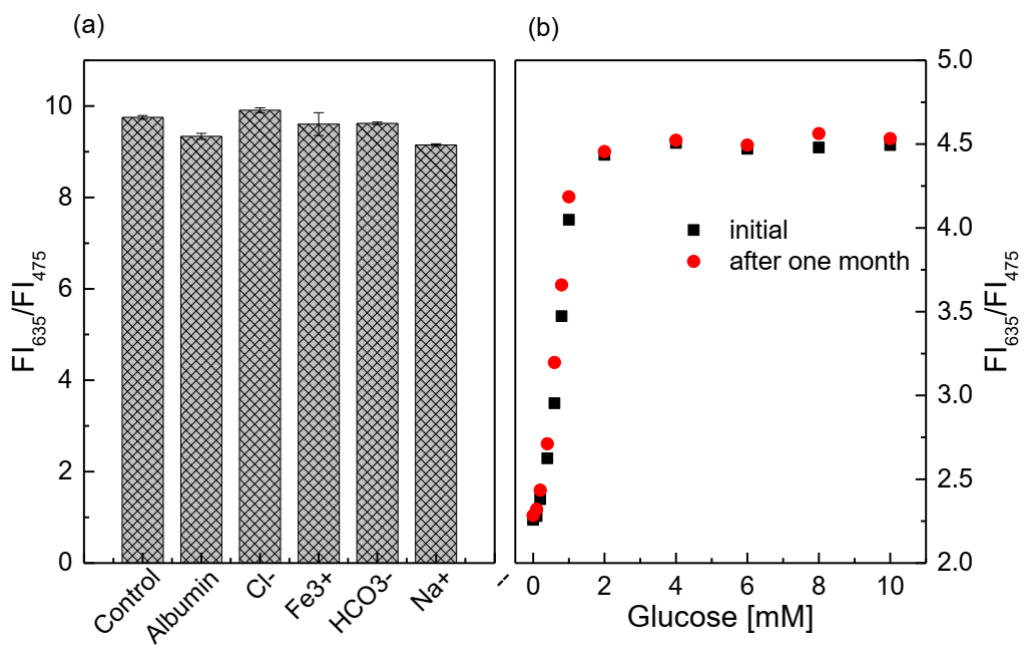
(**a**) Response of the glucose-sensing membrane at 1 mM glucose (control sample), with and without the addition of 145 mM Na^+^, 106 mM Cl^−^, 30 mM HCO_3_^−^, 1.625 mg/L Fe^3+^, and 5 g/dL BSA; (**b**) lifetime of the glucose-sensing membrane at different glucose concentrations.

The presence of interfering substances at high concentration thresholds seems to have a slight effect on the glucose-sensing membrane (Figure 7a), because the deviation of the ratio of FI_635_/FI_475_ between the control sample and the sample containing the interfering substance is approximately 0.2–5%. In addition, the statistical analysis shows that, when comparing the control sample with the sample containing the interfering substance, the *p*-value is always greater than 0.05 (*p*-value > 0.05). This means that the glucose-sensing membrane is less affected by the presence of the above-mentioned factors during the measurement. After 1 month of continuous glucose measurements using the glucose-sensing membrane, the sensitivity of the glucose-sensing membrane was quite high (Figure 7b). The SIs in the glucose concentration range from 0.1 to 2 mM did not change significantly, as the SI was 1.2238 initially and 1.2084 after 1 month of use.

### 3.5. Characterization of the Lactate-Sensing Membrane

**Figure 8:**
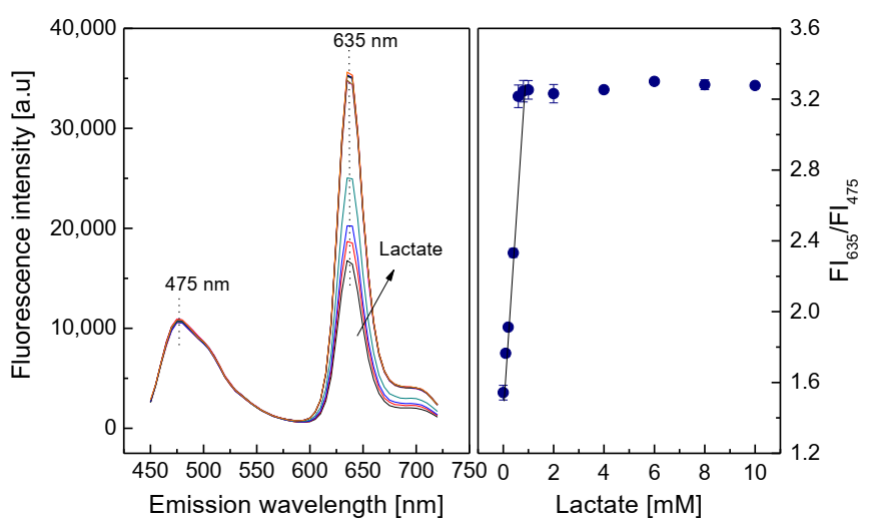
Response of the lactate-sensing membrane at different lactate concentrations, and calibration curve for lactate as determined by ratio of FI_635_/FI_475_.

In the oxidation reaction of lactate, the oxygen consumed and the fluorescence intensity of the lactate-sensing membrane at λ*_em_* = 635 nm both increased with increasing lactate concentrations in the range of 0.1–1 mM (Figure 8). The linear detection range of the lactate-sensing membrane was chosen to be 0.1–0.8 mM, with a limit of detection (LOD) of 0.06 mM. The activity of LOX immobilized on the sensing membrane was evaluated via Michaelis–Menten kinetics. The maximal reaction rate (V_max_) of 1250 mM/min and the Michaelis–Menten constant (K_m_) of 0.375 mM were obtained from the Lineweaver–Burk plot by using the ratio of the emission fluorescence intensities at λ*_em_* = 475 nm and 635 nm. The photos of the response of the oxygen-sensing membrane shown in Figure 2c could be compared with the graphs on the left side of Figure 8 and the oxygen concentration in the detection of lactate could vary in the range of 0–21%.

**Figure 9:**
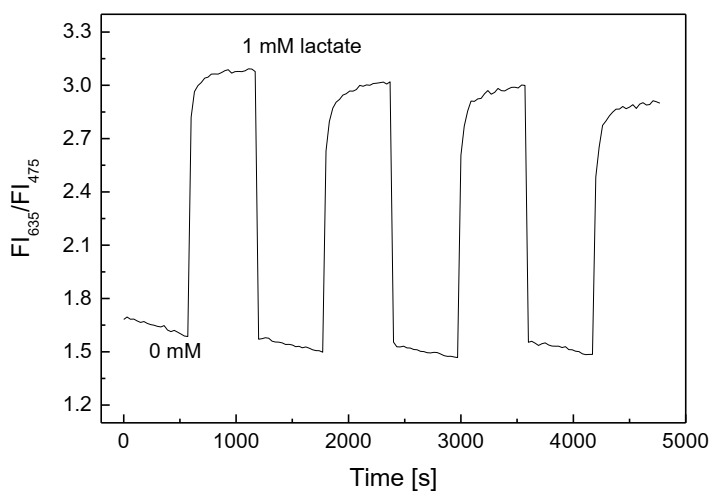
Reversibility of the lactate-sensing membrane repeatedly exposed at 0 and 1 mM lactate.

The reversibility of the lactate-sensing membrane is similar to that of the glucose-sensing membrane. As shown in Figure 9, it is excellent when exposed to a cycle of lactate concentrations of 0 and 1 mM. The lactate-sensing membrane showed fast recovery between 0 and 1 mM lactate, with small values of RSD: 3.55% at 0 mM and 3.69% at 1 mM lactate, even though the fluorescence intensity of the lactate-sensing membrane seemed to decrease after prolonged repeated exposure to 1 mM lactate and distilled water. These results also indicate that the layer containing LOX did not significantly affect the contact between the PS@PtP*Si@C6 membrane and the oxygen in the lactate solution, since the saturation point was reached in the shortest time.

**Figure 10:**
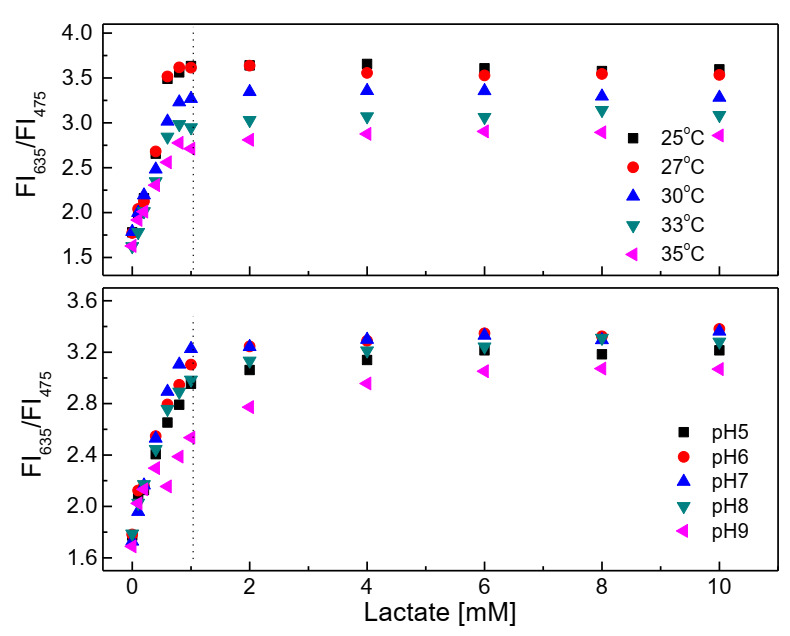
Response of the lactate-sensing membrane at different lactate concentrations in the temperature range of 25–35 °C and pH range of 5.0–9.0.

The response of the lactate-sensing membrane is illustrated in Figure 10 (upper graph) with different lactate concentrations at different temperatures. The figure shows that the sensitivity of the lactate-sensing membrane clearly decreased with increasing temperatures toward 35 °C. In fact, the higher the temperature, the higher the activity of the enzyme, but in this case, the sensitivity of the sensing membrane decreased at high temperature.

As shown in Figure 10 (lower graph), the lactate-sensing membrane showed preference for use in the pH range of 6–8, since the ratio of the fluorescence intensities at *λ_em_* = 475 nm and 635 nm of the lactate-sensing membrane did not change significantly at any lactate concentrations in the range of 0.1–1 mM in this pH range.

**Figure 11:**
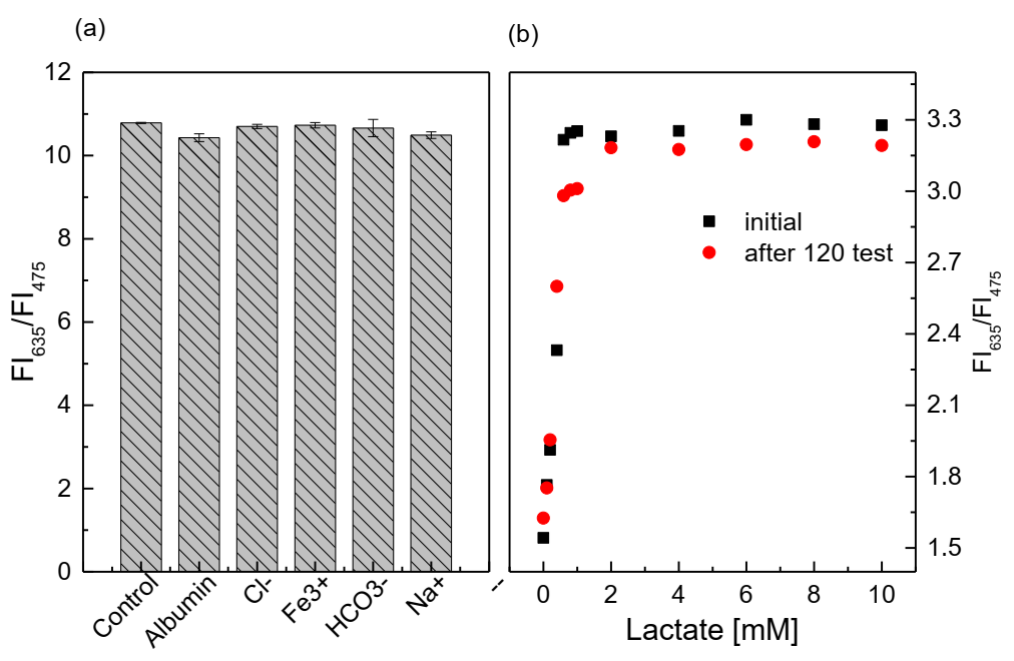
(**a**) Response of the lactate-sensing membrane at 1 mM lactate (control sample), with and without the addition of 145 mM Na^+^, 106 mM Cl^−^, 30 mM HCO_3_^−^, 1.625 mg/L Fe^3+^, and 5 g/dL BSA; (**b**) lifetime of the lactate-sensing membrane at different lactate concentrations.

Similar to the glucose-sensing membrane, the presence of the interfering substances at high concentration thresholds does not appear to significantly affect the performance of the lactate-sensing membrane (Figure 11a), because the deviation of the ratio of FI_635_/FI_475_ between the control sample and the sample containing interference is approximately 0.6–3.3%. Further, according to the statistical analysis, the *p*-values are always larger than 0.05 (*p*-value > 0.05) when comparing the control sample with the sample containing the interfering substance.

After 120 tests of continuous lactate measurements with the lactate-sensing membrane, the sensitivity of the lactate-sensing membrane was decreased by about 16.7% (Figure 11b). The SIs in the lactate concentration range of 0.1–0.8 mM changed significantly, since the SI varied from SI_initial_ = 2.34 to SI_120test_ = 1.95. This contributed to the shorter lifetime of LOX than that of GOD.

### 3.6. Applications of the Glucose- and Lactate-Sensing Membrane

Next, glucose and lactate in artificial human plasma were determined using the glucose- and lactate-sensing membranes developed in this work. According to Q. Yang et al. [26], the normal physiological levels of the components in human plasma include 0.125 mM ascorbic acid, 0.33 mM uric acid, 0.5 mM urea, 0.5 mM glycine, and 0.13 mM acetaminophen.

**Figure 12:**
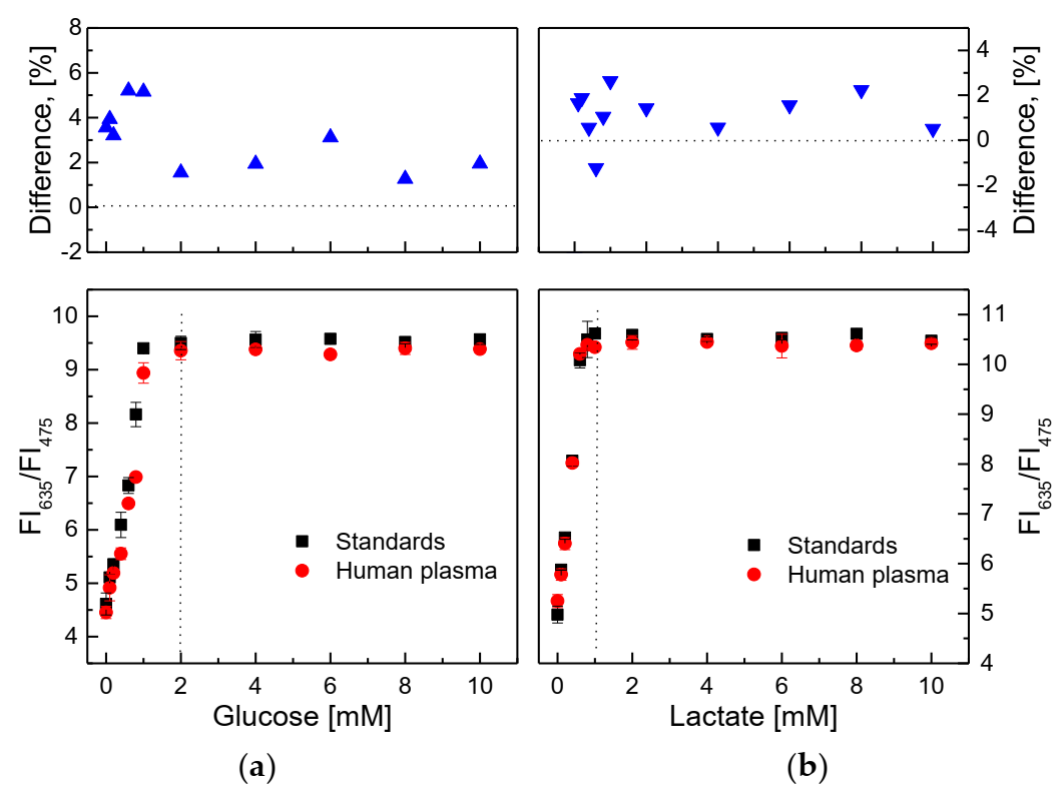
(**a**) Response of the glucose-sensing membrane to solutions of standard glucose and artificial human plasma glucose, and (**b**) response of the lactate-sensing membrane to solutions of standard lactate and artificial human plasma lactate. Percent difference of ratiometric fluorescence intensities (R = FI_635_/FI_475_) in glucose (or lactate) concentrations between standard solution and artificial human plasma represents 100 × {(R_std glucose_-R_human plasma glucose_)/R_std glucose_} or 100 × {(R_std lactate_-R_human plasma lactate_)/R_std lactate_}.

As shown in Figure 12a, the presence of some factors in human plasma solution did not affect the response of the glucose-sensing membrane at different glucose concentrations, since the SIs in the glucose concentration range of 0.1–2 mM were SI_std glucose_ = 2.685 and SI_human plasma glucose_ = 2.639. In the case of the lactate-sensing membrane, Figure 12b shows that the lactate-sensing membrane was well operated under this condition and that the SIs did not change significantly in the lactate concentration range of 0.1–0.8 mM, (i.e., SI_std lactate_ = 7.279 and SI_human plasma lactate_ = 7.126).

An overview on the sensitivity and accuracy of a few biosensors for the determination of glucose and lactate is shown in Table 1. Some analytical parameters were compared with those of the ratiometric fluorescent sensing membranes developed in this work.

A few biosensors based on fluorescence measurements have previously been developed for the determination and monitoring of glucose and lactate in human biofluids, such as tears, urine, blood serum, and sweat. Ratiometric fluorescent sensors can provide a precise and quantitative detection of an analyte, but there has been little research examining the application of ratiometric fluorescent glucose and lactate sensors to study on human health and life. An Eu-metal organic framework (MOF) probe was studied to detect glucose, and it showed high sensitivity, with an LOD of 0.0643 uM [31]. However, it has high synthesis costs and faces some difficulties in repeated use. A ratiometric fluorescent paper sensor based on gold nanoclusters was developed to assess consecutive color change for the visual determination of glucose in the concentration range of 4.4 to 10 mM in human serum [27]. Ratiometric glucose-sensing film was fabricated using an oxygen-sensing layer and a GOD-immobilized layer for the detection of glucose in artificial tears [28]. The oxygen-sensing layer was fabricated through the covalent binding of an oxygen-sensitive dye to poly(2-hydroxyethyl methacrylate)-co-polyacrylamide film, and GOD was immobilized onto the oxygen-sensing film via crosslinking with glutaraldehyde. This glucose sensor showed good analytical performance in terms of sensitivity and stability, as well as its potential application for the detection of glucose in tears. However, the fabrication of an oxygen-sensing film is complicated and expensive. In our previous work [32], we developed a ratiometric fluorescent glucose sensor for glucose detection in tears, wherein dye-doped core-shell particles were used to fabricate an oxygen-sensing membrane. Although the glucose biosensor was sensitive and accurate in determining the glucose concentrations in tears, it was not simple to make core-shell particles doped with both oxygen-sensitive dye and reference dye.

While the biosensors developed in this work are simple to fabricate, they are highly sensitive and stable. The sensing and transducer format can potentially be applied to the development of a few biosensors based on the oxidation reaction in the catalysis of oxidase, such as for ethanol sensors with alcohol oxidase and for urate sensors with urate oxidase.

## 4. Conclusions

In this study, ratiometric fluorescent biosensors for glucose and lactate were successfully developed from the combination of an oxygen-sensing membrane (i.e., the PS@PtP*Si@C6 membrane) with the immobilized enzyme (GOD or LOX). These sensing membranes showed high sensitivity to glucose or lactate in the linear detection range from 0.1 to 2 mM for glucose, and from 0.1 to 0.8 mM for lactate. They also exhibited good selectivity, fast reversibility, and longtime stability. This ratiometric fluorescent sensor has potential as a sensor platform for detecting various analytes in biological fluids, such as human serum, tears, and sweat.

## Figures and Tables

**Table 1:** Comparison of some analytical parameters of glucose and lactate biosensors.

Detection	Method	Linear Detection	LOD	Response Time	Stability	Ref.
Glucose	Amperometry and based on H_2_O_2_ reduction (enzyme + electrode)	0–0.9 mM	0.01 mM	-	-	[5]
	Fluorometry and based on oxygen consumption (enzyme + fluorescence dye)	0–5 g/L (0–28 mM)	0.1 g/L (0.55 mM)	0.15–4.6 min	10 months	[24]
	Fluorometry and based on H_2_O_2_ reduction (enzyme + Au nanoclusters)	1–10 mM	0.16 mM	-	-	[27]
	Fluorometry and based on oxygen consumption (enzyme + fluorescence dye)	0–0.8 mM	0.05 mM	-	7 days	[28]
	Fluorometry and based on oxygen consumption (enzyme + fluorescence dye)	0.1–2 mM	0.031 mM	<1 min	1 month	This work
Lactate	Amperometry (enzyme + electrode)	0–24 mM	-	1 min	-	[16]
	Amperometry (enzyme + electrode)	0.75–1 mM	0.75 µM	-	50 days	[17]
	Fluorometry (enzyme + fluorescence dye)	0.5–10 µM	0.16 µM	6 min	-	[29]
	Fluorometry (enzyme)	1–5 mM	0.45 mM	15 min	-	[30]
	Fluorometry (enzyme + fluorescence dye)	0.1–0.8 mM	0.06 mM	<1 min	120 tests	This work

## Data Availability

Not Applicable.
